# Electrophysiological Evidence Reveals the Asymmetric Transfer from the Right to Left Hemisphere as Key to Reading Proficiency

**DOI:** 10.3390/brainsci13040621

**Published:** 2023-04-06

**Authors:** Sangyub Kim, Joonwoo Kim, Kichun Nam

**Affiliations:** 1 Wisdom Science Center, Korea University, Seoul 02841, Republic of Korea; sangyub0310@gmail.com; 2 Department of Psychology, Korea University, Seoul 02841, Republic of Korea; psymon@korea.ac.kr; 3 School of Psychology, Korea University, Seoul 02841, Republic of Korea

**Keywords:** visual word recognition, interhemispheric interaction, word familiarity, granger causality, electrophysiological activity, N100, N400

## Abstract

The present investigation aimed to explore the interhemispheric interactions that contribute to changes in reading proficiency by examining the processing of visual word recognition in relation to word familiarity. A lexical decision task was administered to 25 participants, and their electrophysiological activity was recorded. A behavioral analysis showed the faster and more accurate processing of highly familiar words compared to less familiar ones. An event-related potential analysis uncovered an asymmetric familiarity effect over the N100 and N400 components across the two hemispheres, indicating an asymmetrical word familiarity processing. Granger causality analyses demonstrated a stronger transfer of information from the right hemisphere (RH) to the left hemisphere (LH) during the N100 processing and a weaker transfer from the LH to the RH during the N400 processing for highly familiar word recognition. These findings suggest that the asymmetric coordination between the RH and LH occurs early in visual word recognition and highlight the importance of interhemispheric interactions in efficient visual word recognition and proficient reading.

## 1. Introduction

Numerous studies have investigated the neural changes that occur during the development of cognitive abilities. These investigations have encompassed a range of cognitive proficiencies, including problem solving [1], attentional control [2], and spatial/verbal memory [3,4]. Of particular interest is the development of reading proficiency and the corresponding changes in brain activity that are associated with the processing of visual strings during literacy acquisition [5,6,7]. The cerebral cortex, which is responsible for processing visual characters, appears to be evolutionarily immature, as visual letters have only recently emerged in human history. This raises questions about the dynamic interaction between the left and right hemispheres during the visual letter processing in reading. Understanding the hemispheric mechanism involved in the development of reading proficiency allows us to examine the strategic interactions between the two hemispheres as the reading proficiency improves.

The present study investigated the interhemispheric changes in visual word recognition, using an indicator of word familiarity to address the question of how this familiarity influences the proficiency of visual word processing. Familiarity is a crucial factor in visual word processing and is a closer indicator of processing proficiency than corpus-based frequency. In this regard, Yoshizaki (2001) employed familiarity as an indicator of visual word processing proficiency and found a significantly faster response time in the bilaterally presented condition of a familiar script-form than of an unfamiliar script-form, indicating that the processing proficiency was determined by familiarity with lexical items [8]. Kim, Kim, and Nam (2022) further demonstrated the effect of word familiarity on these bilateral interactions and revealed a significant bilateral facilitation in the most familiar word condition [9]. These previous findings suggest that the interhemispheric coordination in visual word processing is modulated by word familiarity, indicating that visual word recognition proficiency is closely related to changes in the intercommunication between the left and right hemispheres.

Notwithstanding the previous findings, there remain some limitations to the study of the interhemispheric interactions in visual word recognition. Firstly, the majority of previous studies have presented visual words in the parafoveal vision, which is not representative of the typical reading process. During normal reading, visual words are typically placed within the foveal angle to ensure the optimal recognition and efficient propagation of the word information to the brain [10,11]. However, a parafoveal presentation blurs the visual word and is processed at a lower resolution due to the distance of eccentricity. Secondly, while the behavioral marker in the visual half-field paradigm has been used to examine the hemispheric propagation of visual information, it does not provide direct evidence of bi-hemispheric responses, and the causality of these interhemispheric interactions cannot be fully inferred from behavioral data. An electroencephalogram (EEG), on the other hand, is a non-invasive method for measuring the electrical activity of the brain at a high temporal resolution, which can provide direct evidence of the causal response of bi-hemispheric processing. In particular, event-related potentials (ERPs) analyses enable a direct assessment of the hemispheric responses after a stimuli presentation. Therefore, in the present study, visual words were presented in the foveal vision and the electrophysiological responses to this were recorded using an EEG to investigate the effect of word familiarity on the electrical activities of the interhemispheric interactions.

Previous studies have identified an electrophysiological marker for increased interhemispheric cooperation by demonstrating a bilateral redundancy gain (BRG) for words compared to nonwords in visual word recognition [9,12]. Using a tachistoscopic paradigm, Mohr et al. (2007) asked participants to perform a lateralized lexical decision task by presenting a visual word in the unilateral or bilateral visual fields and comparing the ERPs after the stimulus presentation [12]. They observed a significant increase in the amplitude after the word presentation, at 160–200 ms for bilateral presentation relative to unilateral presentation, while no such increase was observed for pseudoword processing. The authors suggested that this increase in the amplitude for words indicated a significant interaction between the lexicality (word vs. pseudoword) and presentation mode (bilateral vs. unilateral presentation), reflecting the summation of neural activation from the distributed lexical pathways of the two hemispheres. Their findings suggest the presence of an interhemispheric cooperation indicator for lexical proficiency at 160–200 ms. Additionally, studies examining frequency effects have reported an early significant increase in amplitude of around 150 ms after word presentation [13,14], although other studies have found frequency effects on amplitudes at various latencies, ranging from 250 ms to 800 ms [13,14,15,16,17,18,19]. These findings indicate the importance of examining familiarity-related ERP components for reading proficiency in the early time window of around 200 ms.

Prior studies have reported the presence of familiarity effects in ERP components, which are anticipated to be asymmetrically expressed in the two hemispheres. Prior research has suggested the presence of an asymmetric transfer from the non-dominant hemisphere to the dominant hemisphere during interhemispheric processing [20], indicating a transfer from the right hemisphere (RH) to the left hemisphere (LH) in the case of language processing. Based on the findings of the bilateral facilitation for the most familiar word condition in Kim et al. (2022) [9], we predict that this asymmetric transfer is likely to be stronger for words with a high familiarity. Furthermore, this facilitative interaction suggests a stronger unidirectional transfer from the RH to the LH than from the LH to the RH. Additionally, we utilized a Granger causality analysis to reveal the pattern of the time-dependent causal interactions between the left and right hemispheres based on the level of word familiarity in the foveal word processing [21]. We expect that the Granger causality analysis will demonstrate a stronger asymmetric transfer from the RH to the LH in the recognition of more familiar words.

Based on prior studies that have investigated the effects of word frequency on the ERP components during the initial stages of visual word processing [13,14], as well as the potential interhemispheric cooperation during the early stages of visual word recognition [12], we postulated that a comparable pattern of effects would emerge in response to word familiarity. Specifically, we hypothesized that hemispheric asymmetry would be elicited from the ERP components by familiar words during the early time window, whereas unfamiliar words would produce more symmetrical effects. Additionally, we anticipated that the effects of familiar words would be characterized by stronger inter-hemispheric interactions and asymmetry across the hemispheres, particularly within the time range of 100–200 ms, in the early stages of visual word processing. To test our research hypothesis, we examined whether this familiarity effect occurred in the behavioral responses and whether word familiarity modulated the interhemispheric interactions by observing the asymmetric familiarity effect in the two hemispheres.

## 2. Methods

### 2.1. Participants

In the current study, 25 individuals of native Korean nationality were enrolled as participants. All the subjects demonstrated a strict adherence to the experimental protocol, yielding a final dataset that was inclusive of all 25 subjects. We evaluated their handedness using the Edinburgh Handedness Inventory [15], showing that all the participants were right-handed (M: 6.64, SD: 2.55). In order to ensure the reliability of the study, strict inclusion and exclusion criteria were employed to recruit the participants. Specifically, individuals were excluded from participation if they met any of the following criteria: (1) a documented history of neurological impairment resulting from a stroke or brain damage, (2) a diagnosis of any psychiatric disorder, (3) an inability to provide voluntary consent, and (4) a pre-existing medical condition that may interfere with their ability to participate in the study. By implementing these rigorous criteria, the study aimed to minimize any potential confounds and ensure the validity of the results. Normal or corrected-to-normal vision was also required for participation. The cohort exhibited an average age of 23.84 years (SD: 2.58 years, range: 20–29 years). The present study was conducted in strict compliance with the ethical principles outlined in the 1964 Declaration of Helsinki. Prior to participation, the subjects were informed of these ethical standards and provided their explicit consent. As compensation for their participation, the subjects received a nominal monetary reward.

The present study employed power analyses to determine the appropriate sample size required to detect significant effects in both their behavioral and electrophysiological responses. The effect sizes in the power analyses of the behavioral and electrophysiological responses were evaluated through the statistical analyses in the current study, which are identified in the results section of this article. Our investigation focused on effect sizes of 0.375 for the reaction times and 0.338 for the accuracy, as well as a type 1 error rate of 0.05 (α), a power of 0.80 (1-β error probability), four levels for the within condition (FAM1, FAM2, FAM3, and FAM4 levels for the familiarity condition), and 75 measurements (the number of stimuli per condition) for the behavioral response power analysis. Through this analysis, we determined that a sample size of eight participants was necessary to detect significant effects. Additionally, we conducted a power analysis using the electrophysiological responses within a targeted time window of N100 (130–210 ms) and N400 (400–500 ms). Here, we considered the 0.124 and 0.137 effect sizes, while utilizing a type 1 error rate of 0.05, a power of 0.80, four levels for the within condition, and 75 measurements for the electrophysiological response power analysis. Our analysis indicated that a sample size of 20 participants was necessary to obtain significant electrophysiological responses. To ensure the detection of significant effects in both the behavioral and electrophysiological responses, we concluded that a sample size of at least 20 participants was required for the current study. All the effect sizes, including those utilized for the power analyses, are reported in the results section of this paper.

### 2.2. Experimental Task and Procedure

The experimental paradigm employed in this study involved a foveal lexical decision task, which all the participants were required to perform. Initially, a fixation point (+) was presented for a duration of 500 ms, after which, arbitrary letter strings were briefly displayed to the central visual field for 180 ms. Following this, an empty black screen was presented for 1500 ms to allow for a subject response. All the stimuli were presented in a random order. Within this period, all the participants were required to determine whether the presented letter strings were words or nonwords by pressing the ‘slash’ key with their right index finger for words, and the ‘z’ key with their left index finger for nonwords, until the appearance of three asterisks (***). The asterisks were displayed for 1500 ms before disappearing. The response keys for the lexical decision were counterbalanced across the participants.

### 2.3. Materials

A corpus of 600 stimuli was utilized in this study, comprising 300 Korean words and 300 pseudowords. The word stimuli were procured from a variety of sources, namely books (30%), newspapers (20%), movies (10%), and online posts or blogs (40%), as has been reported previously [22,23]. Only lexically unambiguous nouns were included in the stimulus set. In contrast, the pseudowords were generated by a random amalgamation of syllables sourced from the noun words that were featured in the stimulus set. Notably, the pseudowords were deemed to be pronounceable and orthographically legitimate, owing to the use of syllables extracted from the words. Consequently, unlike nonwords, which are not easily pronounced and are orthographically illegitimate, the use of pseudowords is likely to circumvent the influence of orthographic legality on the word familiarity during visual word processing.

### 2.4. Experimental Conditions

The present study investigated the influence of word familiarity, which refers to the degree of familiarity one has with a word, on the interhemispheric interactions in the visual half-field paradigm. To assess the word familiarity, we utilized the subjective familiarity ratings that were collected by Kim et al. (2020), using a 7-point scale ranging from 1 (most unfamiliar) to 7 (most familiar) [22]. Kim, Kim, and Nam (2022) previously employed these data to divide words into four familiarity levels to explore the effect of word familiarity on interhemispheric interactions [9]. As they observed a significant impact of word familiarity on bilateral interaction, we adopted their approach and divided the 300 morphologically complex words into four familiarity levels (FAM1: most unfamiliar, FAM2: slightly unfamiliar, FAM3: slightly familiar, and FAM4: most familiar), with 75 words in each level. The means and standard deviations of the familiarity scores for each level were as follows: M = 4.21 and SD = 0.74 for FAM1, M = 4.61 and SD = 0.91 for FAM2, M = 5.10 and SD = 0.75 for FAM3, and M = 5.46 and SD = 0.78 for FAM4 (M: mean, SD: standard deviation). Kim et al. (2022) confirmed the significant main effect of subjective familiarity on words using one-way (familiarity: FAM1, FAM2, FAM3, and FAM4) analyses of variance (ANOVA) [*F*(3, 296) = 35.415, *p* < 0.001] [9], showing a gradual increase in the scores from the FAM1 level to the FAM4 level. In addition, Kim et al. (2022) conducted an evaluation of four physical length variables (the number of strokes, number of phonemes, number of syllables, and number of morphemes), along with two semantic and frequency variables (the number of objective meanings and frequency of the first syllable), sourced from the Korean Sejong Corpus [24]. This was performed in order to match these six lexical variables between the four familiarity levels, as these variables had the potential to impact the familiarity effect in visual word recognition (Table 1).

### 2.5. EEG Data Acquisition

In the present study, both behavioral and continuous EEG data were recorded from 32 Ag/AgCl surface electrodes (Fp1, Fp2, F3, F4, C3, C4, P3, P4, O1, O2, F7, F8, T7, T8, P7, P8, Fz, Cz, Pz, FC1, FC2, CP1, CP2, FC5, FC6, CP5, CP6, TP9, and TP10), based on the 10–20 International System. The BrainAmp amplifier system was used for the signal acquisition, with a sampling rate of 250 Hz and a band-pass filter that ranged from 0.01 to 30 Hz. The impedance of the electrodes was kept below 10 KΩ during the data acquisition. The Brain-Vision recorder was used to record the acquired signals. In addition, the left and right mastoid reference electrodes were employed to obtain an offline average reference montage. To detect eye blinks and reject eye movement artifacts, the EOG was placed below the right eye.

### 2.6. EEG Data Analysis

The EEG data were analyzed using the open-source EEGLAB toolbox in MATLAB, as described by Delorme and Makeig (2004) [25]. The continuous data were digitally filtered offline using a 30 Hz low-pass filter. Epochs of a 900 ms duration were time-locked from 100 ms before the stimulus onset to 800 ms after the onset and included only the correct responses. To reject artifacts such as eye blinks, muscle movements, and other possible artifacts, we employed the Multiple Artifact Rejection Algorithm (MARA), which calculates the artifact probability for independent components, using an independent component analysis (ICA) that is based on machine learning algorithms, as described by Winkler, Haufe, and Tangermann (2011) [26]. In total, 4 of the 25 subjects were excluded from the EEG analysis as they showed a rejection of more than half of the independent components. The data of the remaining 21 subjects showed about a 5% independent component rejection rate. After the artifact rejection, the ERP responses were baseline corrected and averaged across the subjects according to the level of word familiarity. We selected time windows for each ERP component (N100, N250, P300, N400, and P600) to examine the effect of word familiarity on the interhemispheric interactions. The time windows for each component were 130–210 ms for N100, 210–300 ms for N250, 300–400 ms for P300, 400–500 ms for N400, and 500–650 ms for P600. Three-way repeated measures ANOVAs were performed on the average responses in the continuous EEG data at each time window, with Familiarity (FAM1/FAM2/FAM3/FAM4), Hemisphere (Left/Right), and Column (Anterior/Central/Posterior) as the factors. In addition, we employed a correction for the false discovery rate (FDR) at q = 0.05, in order to determine a corrected significance threshold by avoiding the multiple comparison problem [27,28,29].

### 2.7. Granger Causality Analysis

In this investigation, we employed a Granger causality analysis to assess the causal interactions among the electrode regions on the scalp, namely the left anterior (LA): F3, FC1, and FC5; the left central (LC): C3, CP1, and CP5; the left posterior (LP): P3, P7, and O1; the right anterior (RA): F4, FC2, and FC6; the right central (RC): C4, CP2, and CP6; and the right posterior (RP): P4, P8, and O2. The evoked response of each electrode region was obtained as an average of the electrodes that belonged to it. The Granger causality analysis employed lagged vector autoregression models to establish the significance of the specific time-varying signals in predicting other signals in the future, given the possibility of all the signals of the variables being potentially causative in the system [30]. Specifically, we deemed one signal to Granger cause another signal when the error was notably high in the model without the first signal.

Prior to conducting the Granger causality analysis, we rigorously established the necessary conceptual preconditions to differentiate between causation and mere correlation. Firstly, we included all the potential variables that possessed causal associations [31]. As we sought to investigate hemispheric asymmetry and its interplay with the degree of word familiarity, we selected six electrode groups (LA, LC, and LP for the left hemisphere and RA, RC, and RP for the right hemisphere) that were anticipated to exhibit hemispheric causal interactions. Secondly, we applied the requisite statistical assumptions for the Granger causality analysis. Specifically, we required the covariance stationarity of the time courses in electrophysiological activities, which necessitated that the probability distribution of each time series data remained constant over time. To test for unit roots (*p* < 0.01) and address the issue of covariance stationarity, we employed the Augmented Dickey–Fuller (ADF) test. If non-stationarity was detected, we utilized backward differencing to achieve a covariance stationarity until the unit root was eliminated [31].

To prevent overfitting issues, we employed the conventional approach of selecting the lag order in the Granger causality analysis based on the best Akaike model order [32]. This approach enabled us to investigate the key aspects of Granger causality without the problem of overfitting. Specifically, we utilized lagged multivariate vector autoregressions to explore the Granger causality between the distinct ERP signals that displayed a significant asymmetry across the two hemispheres. To achieve this, we considered the ERP components of the six electrode groups, three from each hemisphere, that were expected to show hemispheric causal interactions. These multivariate vector autoregressions included the time-courses not only of the target electrode groups, but also of the non-target electrode groups. Our methodology ensured that we obtained robust estimates of the causal interactions between the examined signals [33].

### 2.8. Apparatus

The visual stimuli were presented to the participants in an electrically and acoustically shielded chamber via an LG monitor displaying RGB colors. Specifically, white stimuli were presented within the foveal vision ($2^{{}^{\circ}}$ horizontal and $1.5^{{}^{\circ}}$ vertical visual angles) on a black background. The presentation of the stimuli was controlled through the E-prime 2.0 professional software (Psychology Software Tools, Inc., Pittsburgh, PA, United States). During the experiment, the participants were seated and their chins were positioned on a chin rest to maintain a fixed distance of 65 cm between their nasion and the monitor. Their responses to the stimuli were collected via a keyboard placed in front of them, which allowed for button presses to be recorded.

## 3. Results

### 3.1. Behavioral Data

Firstly, one-way (Familiarity: FAM1/FAM2/FAM3/FAM4) repeated measure ANOVAs were performed on the response times. These found the significant main effect of familiarity (*F*(3, 60) = 12.014, *p* < 0.001, $\eta_{p}^{2}$ = 0.375). The post hoc test on the main effect of the familiarity condition revealed significantly faster responses in the FAM3 level and FAM4 level compared to the FAM1 level (*p* < 0.001 (FDR corrected) for the comparison between the FAM1 and FAM3 levels; and *p* = 0.003 (FDR corrected) for the comparison between the FAM1 and FAM4 levels), and significantly faster responses in the FAM4 level than in the FAM2 level (*p* = 0.012, FDR corrected). This indicates that faster responses were made in the visual recognition of words with a high familiarity. Secondly, one-way (Familiarity: FAM1/FAM2/FAM3/FAM4) repeated measure ANOVAs were performed on the accuracy. These showed the significant main effect of familiarity (*F*(3, 60) = 10.228, *p* < 0.001, $\eta_{p}^{2}$ = 0.338). The post hoc test on the main effect of the familiarity condition revealed significantly more accurate responses in the FAM4 level compared to the FAM1 level (*p* < 0.001, FDR corrected), indicating that more accurate responses were made in the higher-familiarity word recognition. These response times and accuracy results are shown in Table 2.

### 3.2. EEG Data

#### 3.2.1. Time-Locked ERP Components

**Figure 1:**
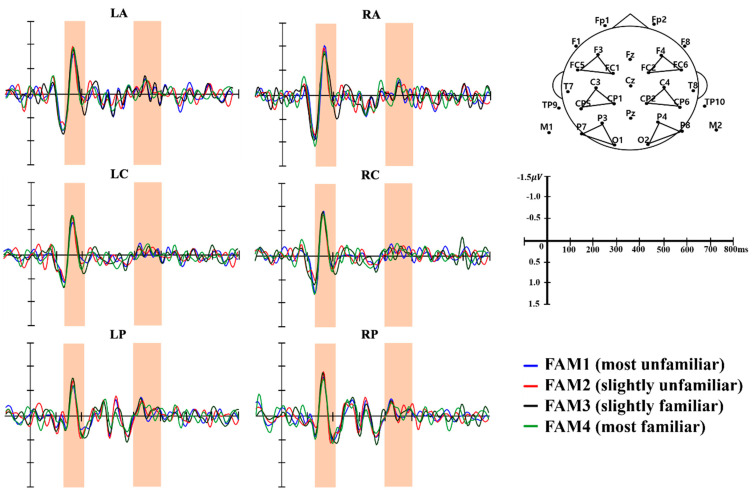
The grand-averaged event-related potentials (ERPs) in response to words of the four familiarity levels at 6 different electrode regions on the scalp. Each grand-averaged line is marked with a different color according to the familiarity level (FAM1−blue line, FAM2−red line, FAM3−black line, and FAM4−green line). The electrode regions were selected as LA (F3, FC1, and FC5), LC (C3, CP1, and CP5), LP (P3, P7, and O1), RA (F1, FC2, and FC6), RC (C4, CP2, and CP6), and RP (P4, P8, and O2). The shaded region in the time window of N100 (130–210 ms) and N400 (400–500 ms) indicates the asymmetric familiarity effect across the two hemispheres (*p* < 0.05, FDR corrected). Abbreviations: LA (left anterior), LC (left central), LP (left posterior), RA (right anterior), RC (right central), RP (right posterior), FAM1 (most unfamiliar), FAM2 (slight unfamiliar), FAM3 (slight familiar), and FAM4 (most familiar).

Three-way (Familiarity: FAM1/FAM2/FAM3/FAM4, Hemisphere: Left/Right, and Column: Anterior/Central/Posterior) repeated measure ANOVAs were performed on each time window of interest within the EEG data (N100, N250, P300, N400, and P600 ERP components). The summary of the grand-averaged ERPs is described in Figure 1.

N100 (130–210 ms). The three-way repeated measure ANOVAs showed the non-significant main effects of the familiarity and hemisphere [*F*(3, 60) = 1.323, *p* = 0.275, $\eta_{p}^{2}$ = 0.062 for Familiarity; *F*(1, 20) = 1.644, *p* = 0.214, $\eta_{p}^{2}$ = 0.076 for Hemisphere], while they showed the significant main effect of the column [*F*(2, 40) = 4.366, *p* = 0.019, $\eta_{p}^{2}$ = 0.179]. The significant main effect of the column indicates stronger N100 amplitudes at the anterior than at the central and posterior (*p* < 0.01 FDR corrected), and at the central than the posterior (*p* < 0.01 FDR corrected). In addition, it showed no significant two-way interaction effects between the familiarity and column (*F*(6, 120) = 0.867, *p* = 0.521, $\eta_{p}^{2}$ = 0.042), and between the hemisphere and column (*F*(2, 40) = 0.068, *p* = 0.934, $\eta_{p}^{2}$ = 0.003), whereas it showed a significant two-way interaction effect between the familiarity and hemisphere (*F*(3, 60) = 2.843, *p* = 0.041, $\eta_{p}^{2}$ = 0.124). A simple main effect analysis for the significant two-way interaction effect between the familiarity and hemisphere revealed the only significant simple main effect of the familiarity at the RH (*p* = 0.052 (FDR corrected) for LH; and *p* < 0.004 (FDR corrected) for RH). The post hoc test for the significant simple main familiarity effect at the RH exhibited the strongest N100 amplitudes at the FAM3 level compared to the FAM1 level (*p* < 0.001, FDR corrected), FAM2 level (*p* = 0.004, FDR corrected), and FAM4 level (*p* < 0.001, FDR corrected). The three-way interaction effect was not significant (*F*(6, 120) = 0.862, *p* = 0.525, $\eta_{p}^{2}$ = 0.041).

N250 (210–300 ms). The three-way repeated measure ANOVAs showed no significant main effects of all the factors (*F*(3, 60) = 2.295, *p* = 0.087, $\eta_{p}^{2}$ = 0.103 for Familiarity; *F*(1, 20) = 2.703, *p* = 0.116, $\eta_{p}^{2}$ = 0.119 for Hemisphere; and *F*(2, 40) = 1.231, *p* = 0.303, $\eta_{p}^{2}$ = 0.058 for Column). In addition, there were non-significant two-way interaction effects between all the factors (*F*(3, 60) = 1.349, *p* = 0.267, $\eta_{p}^{2}$ = 0.063 for between Familiarity and Hemisphere; *F*(6, 120) = 1.035, *p* = 0.406, $\eta_{p}^{2}$ = 0.049 for between Familiarity and Column; and *F*(2, 40) = 1.477, *p* = 0.241, $\eta_{p}^{2}$ = 0.069 for between Hemisphere and Column), and a non-significant three-way interaction effect between all the factors (*F*(6, 120) = 1.146, *p* = 0.340, $\eta_{p}^{2}$ = 0.054).

P300 (300–400 ms). The three-way repeated measure ANOVAs revealed non-significant main effects of all the factors (*F*(3, 60) = 0.357, *p* = 0.784, $\eta_{p}^{2}$ = 0.018 for Familiarity; *F*(1, 20) = 0.064, *p* = 0.803, $\eta_{p}^{2}$ = 0.003 for Hemisphere; and *F*(2, 40) = 0.376, *p* = 0.689, $\eta_{p}^{2}$ = 0.018 for Column). Additionally, they showed non-significant two-way interaction effects between the familiarity and hemisphere (*F*(3, 60) = 1.068, *p* = 0.369, $\eta_{p}^{2}$ = 0.051), and between the hemisphere and column (*F*(2, 40) = 0.487, *p* = 0.618, $\eta_{p}^{2}$ = 0.024), while they exhibited a significant two-way interaction between the familiarity and column (*F*(6, 120) = 3.183, *p* = 0.006, $\eta_{p}^{2}$ = 0.137). A simple main effect analysis for the significant two-way interaction effect between the familiarity and column revealed a non-significant familiarity effect at the anterior column (*p* = 0.732, FDR corrected) and the central column (*p* = 0.380, FDR corrected), whereas it showed a significant familiarity effect in the posterior column (*p* = 0.018, FDR corrected). The post hoc test for the significant simple main effect of the familiarity revealed stronger P300 amplitudes in the more familiar word recognition (*p* = 0.011 (FDR corrected) for the comparison between the FAM1 and FAM4 levels; and *p* = 0.005 (FDR corrected) for the comparison between the FAM2 and FAM4 levels). The three-way interaction effect was not significant (*F*(6, 120) = 1.027, *p* = 0.411, $\eta_{p}^{2}$ = 0.049).

N400 (400–500 ms). The three-way repeated measure ANOVAs revealed non-significant main effects of all the factors (*F*(3, 60) = 0.523, *p* = 0.668, $\eta_{p}^{2}$ = 0.026 for Familiarity; *F*(1, 20) = 3.915, *p* = 0.062, $\eta_{p}^{2}$ = 0.164 for Hemisphere; and *F*(2, 40) = 0.234, *p* = 0.792, $\eta_{p}^{2}$ = 0.012 for Column). In addition, they showed a non-significant two-way interaction effect between the hemisphere and column (*F*(2, 40) = 0.151, *p* = 0.861, $\eta_{p}^{2}$ = 0.007), whereas they exhibited significant two-way interaction effects between the familiarity and hemisphere (*F*(3, 60) = 3.174, *p* = 0.031, $\eta_{p}^{2}$ = 0.137), and between the familiarity and column (*F*(6, 120) = 2.222, *p* = 0.045, $\eta_{p}^{2}$ = 0.100). A simple main effect analysis for the significant two-way interaction effect between the familiarity and hemisphere revealed the significant simple main familiarity effect at the LH (*p* = 0.020, FDR corrected), but not at the RH (*p* = 0.632, FDR corrected). The post hoc test for the significant simple main effect of the familiarity at the LH showed stronger N100 amplitudes in words with the FAM2 level, relative to the FAM4 level (*p* = 0.007, FDR corrected). Additionally, a simple main effect analysis for the significant two-way interaction effect between the familiarity and column showed no significant simple familiarity effect at the anterior column (*p* = 0.070, FDR corrected), the central column (*p* = 0.455, FDR corrected), and the posterior column (*p* = 0.739, FDR corrected). The three-way interaction effect between all the factors was not significant (*F*(6, 120) = 1.047, *p* = 0.399, $\eta_{p}^{2}$ = 0.050).

P600 (500–650 ms). The three-way repeated measure ANOVAs showed non-significant main effects of all the factors (*F*(3, 60) = 0.501, *p* = 0.683, $\eta_{p}^{2}$ = 0.024 for Familiarity; *F*(1, 20) = 0.384, *p* = 0.543, $\eta_{p}^{2}$ = 0.019 for Hemisphere; and *F*(2, 40) = 0.827, *p* = 0.445, $\eta_{p}^{2}$ = 0.040 for Column). In addition, there were also non-significant two-way interaction effects between all the factors (*F*(3, 60) = 0.390, *p* = 0.761, $\eta_{p}^{2}$ = 0.019 for between the Familiarity and Hemisphere; *F*(6, 120) = 0.937, *p* = 0.472, $\eta_{p}^{2}$ = 0.045 for between the Familiarity and Column; and *F*(2, 40) = 0.206, *p* = 0.815, $\eta_{p}^{2}$ = 0.010 for between the Hemisphere and Column), and no three-way interaction effect between all the factors (*F*(6, 120) = 2.013, *p* = 0.069, $\eta_{p}^{2}$ = 0.091).

#### 3.2.2. Granger Causality

**Figure 2:**
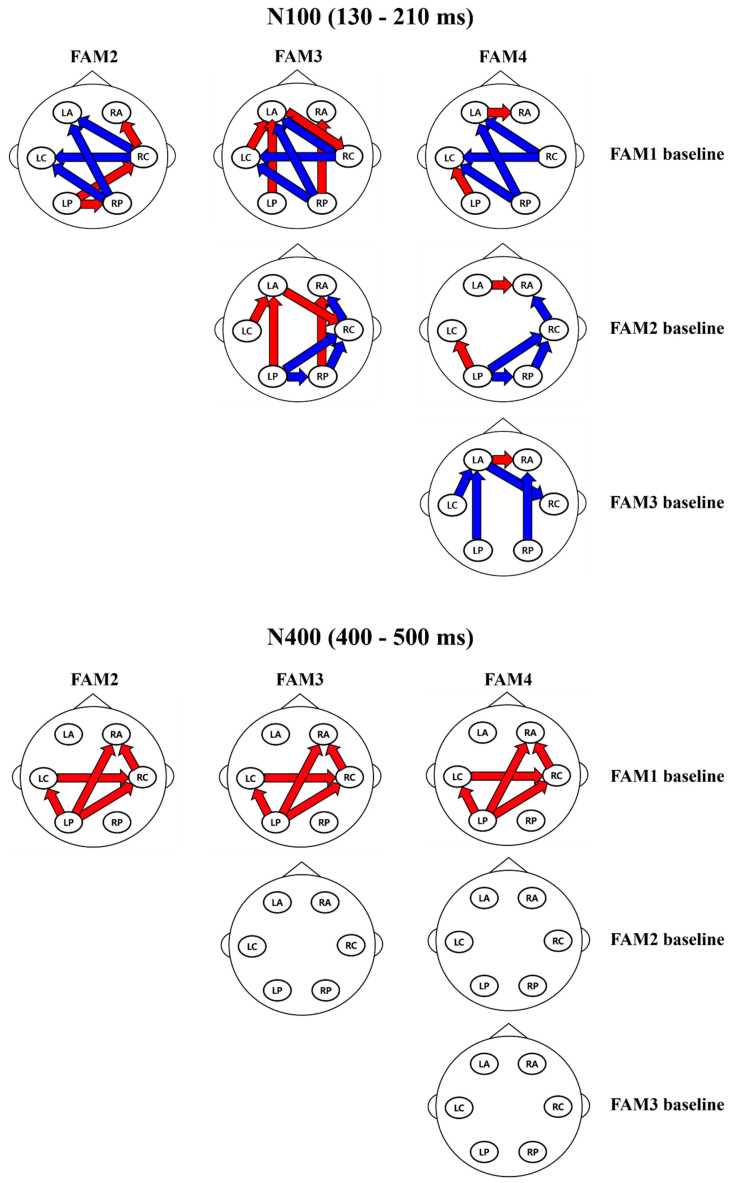
The patterns of the Granger causality between electrode regions on the scalp over N100 (130−210 ms) and N400 (400−500 ms) after the word presentation. This presents the Granger causality in each familiarity level compared to the baselines (FAM1, FAM2, and FAM3). The blue arrows indicate the newly significant Granger causality in the corresponding familiarity level compared to the baselines, while the red arrows denote the Granger causality that changed to insignificant relative to the baselines. Abbreviations: LA (left anterior), LC (left central), LP (left posterior), RA (right anterior), RC (right central), RP (right posterior), FAM1 (most unfamiliar), FAM2 (slight unfamiliar), FAM3 (slight familiar), and FAM4 (most familiar).

Granger causality analyses were conducted to investigate the interhemispheric interactions of the electrophysiological activities on the scalp and examine the interhemispheric alterations depending on word familiarity. The study focused on multiple ERP components that showed hemispheric asymmetry according to the level word familiarity. Specifically, the N100 and N400 components exhibited a significant two-way interaction between the hemisphere and familiarity. Granger causality analyses were performed over the N100 (130–210 ms) and N400 (400–500 ms) time window, and six time-lagged models were selected based on a comparison of the Granger causality model order, ranging from 2 to 20 to describe the primary causality of the Granger network between the two hemispheres [32]. The results of the Granger causality analysis were depicted using blue and red arrows at the baseline familiarity in Figure 2, which shows the significance of each pair between the electrode regions, according to the level of word familiarity. The blue arrows indicate a newly significant Granger causality in the corresponding familiarity level compared to the baseline familiarity. For example, the FAM1 baseline represented the changes in the Granger causality of the visual recognition of words, with FAM2, FAM3, and FAM4 relative to the words with an FAM1 familiarity.

The Granger analyses over N100 revealed a significant Granger causality from the RH to the LH and the extinction of Granger causality from the LH to the RH in the FAM1 baseline analysis, indicating a strong RH–LH asymmetric transfer in the visual recognition of words with a higher familiarity, compared to those with a lower familiarity. Additionally, in the FAM2 baseline analysis, the disappearance of an asymmetric transfer from the LH to the RH was observed in the visual recognition of words with FAM3 and FAM4 compared to FAM2, suggesting that the transfer from the RH to the LH was relatively weakened. Lastly, the FAM3 baseline analysis showed an increased asymmetric transfer from the LH to the RH in the visual recognition of words with FAM4 relative to FAM3. This gradual reduction in the asymmetric transfer from the RH to the LH was attributed to the diminishing familiarity differences between the comparison conditions, i.e., a larger familiarity difference for the comparison between FAM1 and FAM4 than the difference between FAM3 and FAM4.

On the other hand, the Granger analyses over N400 found a significant decrease in the transfer from the LH to the RH for the familiar word recognition. In the FAM1 baseline analysis, there were Granger causalities that became insignificant in the FAM2, FAM3, and FAM4 levels compared to the FAM1 level. Moreover, there were no changes observed in the FAM2 and FAM3 baseline analyses. These results suggest a significant increase in the transfer from the RH to the LH and a significant decrease in the transfer from the LH to the RH in the foveal word recognition, as the word familiarity increased over the N100 and N400 processing, respectively.

## 4. Discussion

This study examined the impact of word familiarity on both the behavioral responses and electrophysiological activities that occur during visual word recognition. The behavioral results demonstrated that more familiar words led to faster and more accurate responses, indicating a familiarity effect. The electrophysiological data revealed an asymmetric familiarity effect between the two hemispheres during N100 and N400 processing, with a significant familiarity effect for N100 over the RH and a significant familiarity effect for N400 over the LH. These findings suggest that word familiarity impacts the interhemispheric interactions during the early stages of visual word recognition. Notably, Granger causality analyses demonstrated a significant asymmetric Granger causation from the RH to the LH during the N100 processing for the visual word recognition with higher familiarity levels (FAM2, FAM3, and FAM4) compared to the lowest familiarity level (FAM1), suggesting an accelerated asymmetric facilitation from the RH to the LH in more familiar word recognition. This asymmetric acceleration between the two hemispheres implies greater co-work of the bilateral hemisphere in familiar word recognition, with a more specified asymmetric transfer from the RH to the LH, leading to more proficient processing in visual word recognition. Additionally, the extinction of the transfer from the LH to RH in the familiar word recognition over the N400 processing may be affected by the emergence of the accelerated transfer from the RH to LH in the early processing of visual word recognition, i.e., N100, as the processing dynamically affects the subsequent processing within/between hemispheres, with a temporal causal relationship. These results reveal a significant alteration in the interhemispheric coordination for visual word recognition, which underscores the changes that accompany the reading and understanding of words.

The present behavioral and electrophysiological investigation focused on the dual-route model (DRC) and its implications for interhemispheric interactions [34,35]. The DRC model posits two processing routes for word recognition: the lexical route, which is sensitive to word frequency and utilizes stored lexical information, and the non-lexical route, which relies on grapheme–phoneme correspondences and is used for unfamiliar words or nonwords. High-frequency words, corresponding to familiar words, are processed through the faster lexical route, resulting in the rapid activation of semantic and phonological representations [13]. In contrast, unfamiliar words or nonwords are processed through the slower non-lexical route [35]. The present study found evidence for the familiarity effects on the N100 component at the RH and on the N400 component at the LH, suggesting the asymmetric effects of word familiarity across the two hemispheres. The Granger causality analyses showed a significant asymmetric facilitation from the RH to the LH during the N100 processing in the visual recognition of words with higher familiarity levels, while it exhibited the extinction of the asymmetric transfer from the LH to the RH during the N400 processing. These findings support the idea that word familiarity affects visual recognition at the early stage of processing, which is consistent with the DRC model. Additionally, the DRC model suggests that words are processed differently depending on their familiarity level, with a more proficient processing for familiar words, which could lead to changes in the pattern of the interhemispheric interactions.

In addition, the present study found evidence supporting the idea that was proposed by Nowicka and Tacikowski (2011) regarding the direction of the asymmetric transfer in language processing [21]. Specifically, they suggested that the lateralization in the LH for language processing may originate from the asymmetrical facilitative transfer from the nondominant hemisphere (RH) to the dominant hemisphere (LH), which is in line with the current study’s findings. Nowicka and Tacikowski (2011) argued that the stronger asymmetric transfer from the RH to the LH in language processing may indicate a greater left-lateralization of the visual processing of words with a high familiarity [21]. Furthermore, previous evidence has suggested that hemispheric lateralization may be achieved by the corpus callosum, a structural pathway that connects the two hemispheres, enabling them to interact with each other in a facilitatory or inhibitory manner, potentially leading to hemispheric lateralization [36,37]. Thus, the asymmetric transfer from the RH to the LH observed in the present study, for familiar word recognition in normal populations with an intact corpus callosum, may be associated with the emergence of left-lateralization for reading.

Behavioral and electrophysiological studies provide support for the familiarity effect in the early time window of visual word processing, specifically at the N100 stage, as demonstrated in this study [12,38,39]. Previous research has estimated that a written word activates the perisylvian areas in the LH around 150 ms after the word presentation, which are involved in the subsequent form analysis and its lexico-semantic processing of the word [39]. Electrophysiological evidence of the N160 component suggests a possible differential effect of the word familiarity in the early time domain, particularly between words in different lexico-semantic categories [12]. Furthermore, bilateral word processing involves the left and right temporal cortex, with memory circuits potentially contributing to the processing of familiar stimuli in bihemispheric processing [12]. The involvement of memory circuits over both hemispheres in foveal familiar word processing co-activates the pathway of the two hemispheres towards lexical entries. The asymmetric transfer from the RH to the LH during bihemispheric processing could activate these lexical entries using the memory circuits across the two hemispheres. The present study found a stronger transfer from the RH to the LH in higher familiar word recognition, which suggests that familiar words may more strongly activate the memory circuits for visual recognition, leading to faster and more accurate behavioral responses in higher familiar word recognition.

Furthermore, the present study contributes to the growing body of evidence supporting bilateral cooperation models of interhemispheric processing [40]. Such models posit that the co-work of both hemispheres leads to a facilitation in visual word processing, resulting in faster and more accurate responses under the conditions of bilateral activation. Allen (1983) proposed two possible mechanisms to account for this facilitation [40]. The first suggests that each hemisphere has unique capabilities for processing visual words, and so compensatory processing from the opposite hemisphere supplements the processing of the other. The second mechanism proposes an interactive model in which both hemispheres share the processing route, thus working together to improve the overall performance. The findings of the present study appear to support the first mechanism, as we observed an asymmetric interaction between the hemispheres in the visual recognition of highly familiar words. Had the second mechanism been at play, we would have expected a bidirectional transfer regardless of the word familiarity. Our results suggest that the co-work of both hemispheres is more likely due to compensatory processing, rather than shared information processing. Thus, the asymmetric facilitation from the RH to the LH that was observed in our study may be associated with the compensatory work for the early processing of visual word recognition.

In addition, Coles and Goldstein (1985) explored the changes in the interhemispheric interactions that occur with different levels of language proficiency during reading by analyzing EEG (electroencephalogram) patterns [41]. They found that electrophysiological activations towards the left occipital regions significantly increased when the reading materials were within the reading ability of the subjects, indicating a left-lateralized processing during more proficient reading. Minagawa-Kawai, Cristià, and Dupoux (2011) proposed a complex model emphasizing the importance of the early perceptual asymmetries in the acoustic characteristics that lead to an increased lateralization to the LH [42], which is responsible for processing phonemes in the mother language. Conversely, the RH is specialized in capturing prosody. Furthermore, they suggested that functional asymmetry tends to become stronger with aging in the native language, indicating that experience of learning can impact the hemispheric asymmetry in a way that is distinct from biological maturation or genetic origins. Bishop, Hardiman, and Barry (2012) showed that children with a language impairment in their phonological processing displayed dispersed responses throughout the bilateral hemispheres for speech sounds, whereas normal children with no language development problems showed left-lateralized focal responses [43]. These findings suggest that the increase in the asymmetric transfer from the RH to the LH as words become more familiar may induce cerebral asymmetry in normal language development. Therefore, left-lateralized processing and the accompanying changes in the interhemispheric interactions between the two hemispheres may be factors in inducing such cerebral asymmetry in normal language learning.

Thus, the present study’s findings suggest a potential association between the observed interhemispheric asymmetric transfer pattern and reading difficulties caused by reading dysfunction, such as dyslexia. The increase in the RH-to-LH asymmetric transfer may lead to a higher reading proficiency, while the absence of this transfer could result in dyslexic symptoms. Previous research on children with dyslexia has shown hyperactivation in various brain regions, such as the right superior frontal gyrus during a narrative comprehension task [44], the left middle/superior temporal gyri and bilateral insula during sentence comprehension [45], and the left fusiform gyrus (visual word form area) during a reading task [46]. These findings suggest that children with dyslexia may have an abnormal pattern of interhemispheric interactions during their visual word processing, which is not seen in typical reading development. Therefore, the biologically determined mechanisms underlying the asymmetric exchanges that were found in this study may play a crucial role in the functional development and associated phenotypes for reading, with a failure in these mechanisms potentially contributing to dysfunctional developments.

For this reason, the observation of a right visual field advantage for visual word recognition potentially indicates a close association with left-lateralization [9,24,47]. This left-lateralization may be due to the asymmetric transfer from the RH to the LH, creating a superior performance for the visual word recognition in the right visual field/left hemisphere compared to the left visual field/right hemisphere. In contrast, face processing has been found to exhibit a left visual field advantage [48,49], possibly due to the maturation of brain regions, with an asymmetric transfer from the left to the right hemisphere. Specifically, Yovel et al. (2003) found an N170 that was larger over the right than the left occipito-temporal region for face processing, indicating early structural face processing and suggesting asymmetric processing across the hemispheres [48]. Moreover, the asymmetries of the N170 correlated positively with the differences in the responses to a unilateral face presentation to the left and right visual fields, particularly in the time windows of 220–280 ms and 400–600 ms. This indicates that an increased hemispheric asymmetry during the initial stages of face processing is linked to a greater advantage for processing faces in the left visual field during the subsequent processing stages. The observed asymmetric processing in the early time window implies the presence of an asymmetric interaction between the two hemispheres for efficient face processing in a neurologically intact brain. Hence, the results indicating an asymmetric interaction between the two hemispheres in visual word and face processing imply that an asymmetrical exchange might be necessary for neural maturation, leading to efficient processing.

Furthermore, the present investigation revealed an interhemispheric pattern of asymmetric transfer, encompassing the parietal and frontal regions, as depicted in Figure 2. While the changes in the processing of the visual cortex are commonly implicated in reading proficiency [50,51,52], recent studies have indicated that the development of reading is also associated with the maturation of brain regions beyond the visual cortex [53]. Specifically, Ionta (2021) conducted a comprehensive review of previous neuropsychological evidence [53], highlighting the importance of visuo–motor integration skills and proposing that reading development may induce neurally based alterations in brain areas other than the visual cortex. Previous research has presented a visual neuropsychological model that explains two main streams [53,54], the “what” pathway in the ventral stream and the “where” pathway in the dorsal stream. The former passes signals from V1 (primary visual cortex), V2 (secondary visual cortex), V3 (third visual cortex), and V4 (fourth visual cortex) up to the inferior temporal cortex, which is primarily engaged in object recognition, while the latter passes signals from V1, V2, V3, and the superior/medial temporal sulcus up to the parietal cortex, which is mainly involved in encoding the visuo–spatial and motion-related processing of a visual input [55,56]. As these two pathways may exhibit distinct patterns of brain activity, it is plausible that reading proficiency improvements may lead to changes in the functional interconnections between brain areas other than the visual cortex.

The present study offers two noteworthy implications. Firstly, it introduces a novel, experimental paradigm of presenting stimuli in the foveal vision, in contrast to prior investigations that have employed the visual half-field paradigm. For instance, Kim et al. (2022) demonstrated the facilitative interactions between the two hemispheres by presenting words in the parafoveal vision [9]. However, due to the behavioral evaluation limitation of the visual half-field paradigm, they could not identify the asymmetric transfer between the two hemispheres. In contrast, the present study provides electrophysiological evidence for this asymmetric transfer by presenting stimuli in the foveal vision, mimicking the normal reading process and enabling a more robust interpretation of the interhemispheric interaction changes. Secondly, we utilized a Granger causality analysis of the electrophysiological activities over both hemispheres to uncover the interhemispheric interaction patterns. Given that the electrophysiological activities were time series data, the Granger causality analysis could effectively reveal the hemispheric dynamics. Thus, employing this analysis on electrophysiological activity is expected to provide a more comprehensive understanding of the causal interactions between the two hemispheres.

## 5. Conclusions

The present study sheds light on the asymmetrical interhemispheric interactions underlying visual word recognition. The results of the lexical decision task reveal that highly familiar words are processed faster and more accurately, and that this effect is associated with the asymmetric pattern of the N100 and N400 processing across the two hemispheres. Additionally, a Granger causality analysis demonstrates the asymmetric transfer of information between the two hemispheres during the N100 and N400 processing, with a stronger transfer from the RH to the LH during the former, and a weaker transfer from the LH to the RH during the latter. These findings highlight the importance of interhemispheric interactions in reading proficiency and suggest that an asymmetric transfer may contribute to the left-lateralized reading proficiency that is observed in individuals. In sum, this study provides valuable insights into the neural mechanisms underlying visual word recognition and the role of hemispheric interactions in this cognitive process.

## Figures and Tables

**Table 1:** The matched lexical variables between levels of word familiarity. The bracket values indicate standard deviations. Abbreviations: FAM1 (most unfamiliar), FAM2 (slight unfamiliar), FAM3 (slight familiar), and FAM4 (most familiar).

	Physical Length Variable				Frequency Variable	Semantic Variable
	# of Morphemes	# of Syllables	# of Phonemes	# of Strokes	First Syllable Frequency (Log)	# of Objective Meanings
FAM1	2.097	3.293	8.213	19.427	3.761	1.347
	(0.336)	(0.487)	(1.509)	(4.919)	(0.485)	(0.688)
FAM2	2.081	3.24	8.053	18.96	3.677	1.653
	(0.302)	(0.566)	(1.643)	(4.458)	(0.581)	(1.797)
FAM3	2.12	3.187	8.093	19.187	3.745	1.493
	(0.327)	(0.456)	(1.307)	(4.096)	(0.412)	(1.07)
FAM4	2.093	3.093	7.893	17.76	3.73	1.507
	(0.293)	(0.597)	(1.737)	(4.526)	(0.468)	(1.095)

**Table 2:** The behavioral performances of words with the four familiarity levels and pseudowords. The bracket values indicate standard deviations. Abbreviations: FAM1 (most unfamiliar), FAM2 (slight unfamiliar), FAM3 (slight familiar), and FAM4 (most familiar).

	Pseudowords	FAM1	FAM2	FAM3	FAM4
RTs (ms)	536 (49)	533 (48)	522 (47)	508 (48)	504 (40)
ACC (%)	0.903 (0.089)	0.924 (0.081)	0.937 (0.081)	0.941 (0.072)	0.959 (0.039)

## Data Availability

The original contributions presented in the study are included in the article. The collected and analyzed data in the present study are available after permission of the authors in case of acceptable requests.
